# Biocatalytic Strategy for Grafting Natural Lignin with Aniline

**DOI:** 10.3390/molecules25214921

**Published:** 2020-10-24

**Authors:** Sabina Gabriela Ion, Teodor Brudiu, Anamaria Hanganu, Florentina Munteanu, Madalin Enache, Gabriel-Mihai Maria, Madalina Tudorache, Vasile Parvulescu

**Affiliations:** 1 Department of Organic Chemistry, Biochemistry and Catalysis, University of Bucharest, Soseaua Panduri 90, sector 5, 050663 Bucharest, Romania; ion.sabina.gabriela@chimie.unibuc.ro (S.G.I.); brudiuteodor@gmail.com (T.B.); vasile.parvulescu@g.unibuc.ro (V.P.); 2 Institute of Organic Chemistry C. D. Nenitescu of Romanian Academy, 202B Spl. Independentei, 060023 Bucharest, Romania; Anamaria.hanganu@unibuc.ro; 3 Department of Technical and Natural Sciences, Aurel Vlaicu University of Arad, Bd. Revolutiei 77, 310130 Arad, Romania; florentina.munteanu@uav.ro; 4 Institute of Biology Bucharest of the Romanian Academy, Splaiul Independentei 296, 060031 Bucharest, Romania; madalin.enache@ibiol.ro (M.E.); gabriel.maria@ibiol.ro (G.-M.M.)

**Keywords:** grafting bioprocess, lignin, peroxidase enzyme, biocatalysis, amino-functionalization

## Abstract

This paper presents an enzyme biocatalytic method for grafting lignin (grafting bioprocess) with aniline, leading to an amino-derivatized polymeric product with modified properties (e.g., conductivity, acidity/basicity, thermostability and amino-functionalization). Peroxidase enzyme was used as a biocatalyst and H_2_O_2_ was used as an oxidation reagent, while the oxidative insertion of aniline into the lignin structure followed a radical mechanism specific for the peroxidase enzyme. The grafting bioprocess was tested in different configurations by varying the source of peroxidase, enzyme concentration and type of lignin. Its performance was evaluated in terms of aniline conversion calculated based on UV-vis analysis. The insertion of amine groups was checked by ^1^H-NMR technique, where NH protons were detected in the range of 5.01–4.99 ppm. The FTIR spectra, collected before and after the grafting bioprocess, gave evidence for the lignin modification. Finally, the abundance of grafted amine groups was correlated with the decrease of the free –OH groups (from 0.030 to 0.009 –OH groups/L for initial and grafted lignin, respectively). Additionally, the grafted lignin was characterized using conductivity measurements, gel permeation chromatography (GPC), thermogravimetric analysis (TGA), temperature-programmed desorption (TPD-NH_3_/CO_2_) and scanning electron microscopy (SEM) analyses. The investigated properties of the developed lignopolymer demonstrated its disposability for specific industrial applications of derivatized lignin.

## 1. Introduction

Lignin was mentioned for the first time as lignum (Latin word for wood) by the Swiss botanist A.P. de Candolle in the 19th century [1]. It was characterized from the beginning as a fibrous biomass (10–25% lignin in biomass) insoluble in water/alcohol and soluble in weak alkaline solutions. Over the time, the research interest in lignin increased exponentially, especially due to its widespread nature. Today, as the second most abundant component of biomass (next to cellulose/hemicellulose), lignin is considered as an important renewable carbon source [2]. Additionally, the paper pulp industry provides lignin as a waste byproduct, which is quite underutilized [3].

**Figure 1:**
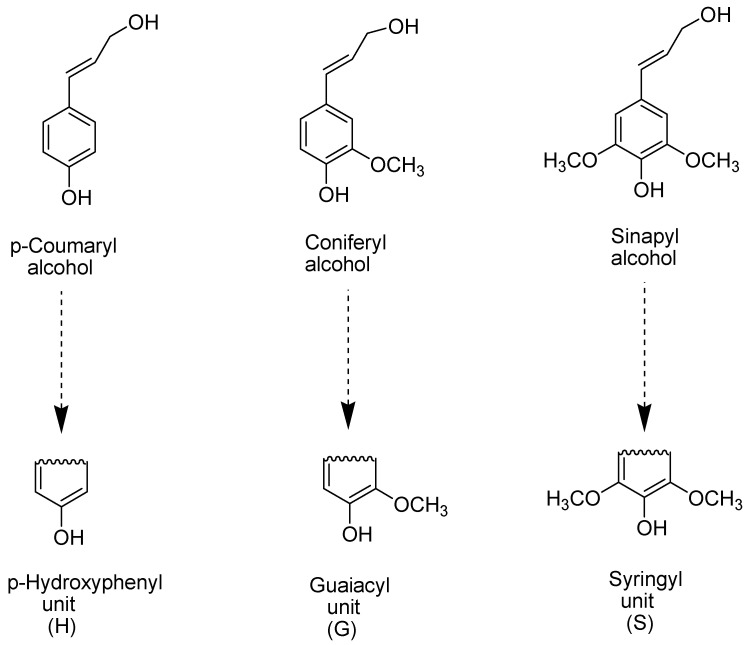
Building blocks and aromatic constituents of lignin.

From the chemical point of view, lignin is a complex heterogenous biopolymer with a 3D structure, which contains phenylpropane units with/without one or two methoxy groups in ortho positions toward an oxygen attached directly to the aromatic ring [4]. It is considered that *p*-coumaryl, coniferyl and sinapyl alcohols, also known as methoxylated hydroxycinnamyl alcohols, represent the monomers of lignin (monolignols) (Figure 1). These are incorporated in different proportions into the lignin structure as three aromatic constituents, namely *p*-hydroxyphenyl (H), guaiacyl (G) and syringyl (S), and are generically called phenylpropan units [5]. The ratio between these units depends on different natural factors, such as the species of plants, climate, geographic area or extraction process [6].

From the structural point of view, phenylpropane units can be connected together through specific interunit linkages, such as β-*O*-4, α-*O*-4, β-5, 5-5’, 4-*O*-5, β-1 or β-β [7]. Therefore, lignin exhibits a large variety of polymeric structures related to the content of the specific interunit linkages and their connection alternatives [8]. Lignin is characterized by a heterogeneous polymeric structure, ensuring in this way important functions in plant cells, such as structural integrity conferring rigidity and resistance to cell walls and transporting water through the vascular system of plants [9]. Enzymatic etherification of the –OH group of lignin monomers initiates the polymerization process [10]. Reactive phenoxy radicals are generated as resonance structures involved in the coupling reaction, leading to dimers linked through ether and C-C bonds. This new dimeric structure could then react with another similar structure or with a monomer, generating trilignols/tetralignols [10]. The final macrostructure yielded by random coupling of the new oligomers is a 3D polymer with different applications [10].

Due to the presence of reactive –OH groups, the lignin structure can be modulated using different processes such as esterification [11], etherification [12], silanization [13], alkylation [14] or grafting [15,16,17,18]. Accordingly, lignins with new properties can be produced. In this way, their homogeneity/heterogeneity, mechanical properties, reactivity and solubility can be improved [19]. The rigidity and resistance could also be conveniently modulated by introducing an anhydride group in the lignin structure [20].

Grafting approach represents another promising alternative to modify the chemical structure of lignin [21,22,23,24,25,26,27,28]. Several grafting strategies taking the –OH groups as targets considered sulfonation [22] and epoxidation [23], leading to materials with antioxidant or antibacterial properties. Halogenation of lignin led to changes in the hydrophobicity/hydrophilicity properties [24], while lignin grafting using ring-opening polymerization of lactide (LA) led to lignin-*g*-poly (lactic acid) co-polymers used as dispersion bio-based composite modifiers [15]. Additionally, kraft lignin was phosphorylated at room temperature with phosphorus pentoxide (P_2_O_5_) in THF (tetrahydrofuran) for improving its thermal stability and was used further as a flame retardant [25]. For phenolic resins, the phenolation process of lignin improved its thermal stability and mechanical properties [26]. Phenolated lignin can be used for the production of formaldehyde resins with adequate curing time and viscosity required for panels [21]. Carboxylation, esterification and alkylation are also valuable chemical strategies for lignin derivatization [21]. Finally, amination as a grafting process of lignin can be used in order to enhance the charge density or/and molecular weight of lignin [27,28]. Therefore, the grafting process allows developing a large lignopolymeric material with improved physicochemical properties. To-date, the main aim of lignin amination was to generate anion-exchange resins, cationic surfactants, flocculants, coagulants and heavy metal adsorbents [29,30,31,32].

Usually, the grafted lignin exhibited suitable properties enabling its use as a raw material for the production of value-added chemicals and materials [15]. Therefore, it can be introduced in mixtures of natural (proteins, starch) or synthetic (polyolefines, polyesters) polymers in order to decrease the melting point and/or increase the crystallization temperature [33]. It can also be used in the construction of adsorbent materials (e.g., aniline removal from waste water) [34], rod-shape porous carbon for use as electrical double layer capacitors (EDLC) in electrode materials [35] or support for the immobilization of lipase enzyme [36]. The modification of the structure with polyaniline led to changes in polymer dispersibility and conductivity [37]. Since a large variety of the industrial applications that are available today are requesting derivatized lignin with predefined properties, searching for new alternatives of grafting lignin is of great interest. Additionally, new grafting methods avoiding the drawbacks of conventional organic chemistry are always welcome for green and sustainable chemistry. Biochemical (enzymatic) strategies could be good alternatives and are currently unexplored in the area of grafting lignin.

**Scheme 1:**
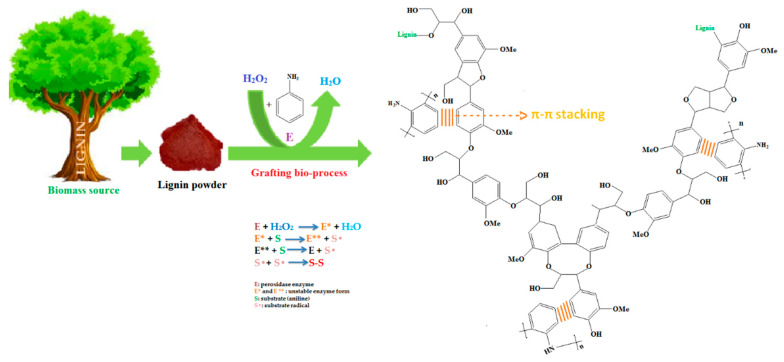
Grafting lignin with aniline using enzymatic biocatalysis.

In this context, we developed a biocatalytic alternative for the derivatization of lignin based on a grafting approach (grafting bioprocess) using aniline as the co-monomer (Scheme 1). The insertion of aniline in the lignin structure was performed using peroxidase enzyme as the catalyst and H_2_O_2_ as the oxidation reagent. The monitoring of the enzymatic process was carried out based on UV-vis and Folin-Ciocalteu (F-C) analyses. FTIR and NMR techniques allowed identifying the presence of the amine groups in the grafted lignin. Additionally, the grafted lignopolymeric material was characterized by performing gel permeation chromatography (GPC), thermogravimetric analysis (TGA) and conductivity, temperature-programmed desorption (TPD-NH_3_/CO_2_) and scanning electron microscopy (SEM) analyses. Therefore, the reported study offers a detailed overview on biografting lignin with aniline following two aspects: (i) the performance of the enzymatic process under different experimental conditions and (ii) the characteristics of the grafted lignin related to the original lignin.

## 2. Results and Discussion

### 2.1. Grafting Bioprocess of Lignin

**Figure 2:**
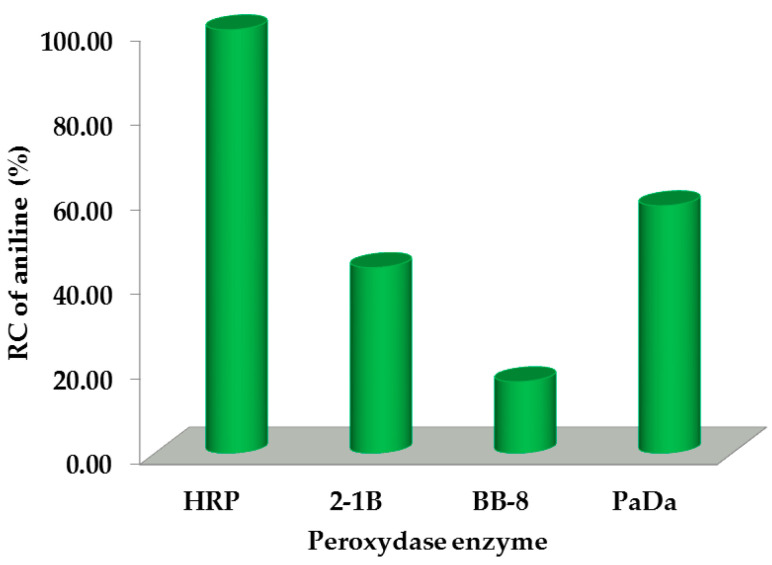
Enzyme screening for the grafting process of lignin with aniline. Experimental conditions: 21.70 mg/mL AL, 23.30 mM aniline, 192 mM H_2_O_2_ (30%, *w*/*w*), 11% (*v*/*v*) peroxidase enzyme (2.5 U mg^−1^ peroxidase activity) and 22% MeOH in PBS (10 mM, pH 7.4); 24 h, 40 °C and 1000× *g* rpm.

The screening of peroxidase enzymes was performed by testing the biocatalytic capacity of the peroxidase from different sources for the grafting of lignin (AL) with aniline (Figure 2). The performance of the biocatalytic process was evaluated based on aniline conversion (aniline attached/inserted to the lignin polymer). In the investigated series (HRP, 2-1B, BB-8 and PaDa), HRP exhibited the highest catalytic capacity of aniline insertion (Figure 2). In contrast, BB-8 peroxidase afforded only a very poor insertion (Figure 2). It was noticed that the peroxidase enzymes exhibited similar enzyme activity under the experimental conditions (around 2.5 U mg^−1^ peroxidase activity). Hence, HRP was chosen for subsequent experiments.

**Figure 3:**
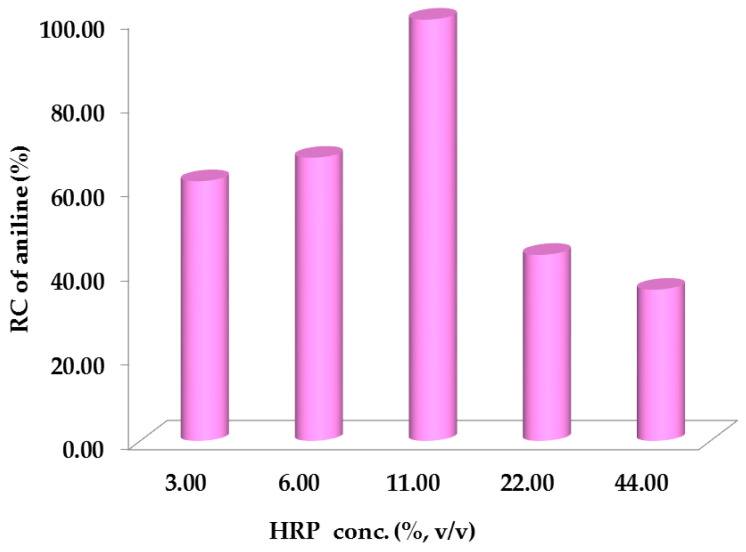
The influence of the concentration of the biocatalyst on the grafting bioprocess. Experimental conditions: 21.70 mg/mL AL, 23.30 mM aniline, 192 mM H_2_O_2_ (30%, *w*/*w*), HRP enzyme and 22% MeOH in PBS (10 mM, pH 7.4); 24 h, 40 °C and 1000× *g* rpm.

Figure 3 presents the effect of HRP concentration on the grafting bioprocess. Concentrations in the range of 3–11% HRP entailed an increase in aniline conversion, where the positive trend of the conversion can be explained by the ascending number of catalytic sites accompanying the HRP concentration. However, larger concentrations (22% and 44% HRP) caused a decrease of the process efficiency that could be assigned to enzyme agglomeration in the reaction phase, leading implicitly to a blockage of the enzyme catalytic sites. Based on these results, the following experiments considered a concentration of 11% HRP.

**Figure 4:**
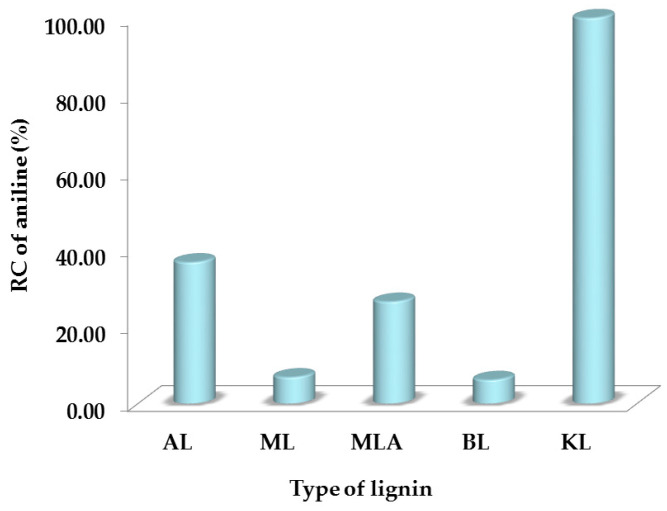
Tests with different types of lignin in the grafting bioprocess. Experimental conditions: 21.70 mg/mL lignin, 23.30 mM aniline, 192 mM H_2_O_2_ (30%, *w*/*w*), 11% (*v*/*v*) peroxidase enzyme (2.5 U mg^−1^ peroxidase activity) and 22% MeOH in PBS (10 mM, pH 7.4); 24 h, 40 °C and 1000× *g* rpm.

Figure 4 shows experimental results for grafting different lignins. KL showed satisfactory conversion, while ML and BL limited the insertion of aniline (<7% relative conversion). The grafting availability of KL lignin is provided by the large abundance of the etheric bounds and high stability of the lignin structure [38]. In contrast, lignins extracted via acidic approaches exhibited only low conversion in the grafting bioprocess. This behavior could be explained by the large density of free –OH groups, which favored intra oxi-polymerization of lignin against its interaction with the aromatic amine. Consequently, intra polymerization of lignin produced lignin grafted with aniline. The approach utilized for lignin extraction from biomass affected the lignin structure and, furthermore, the grafting bioprocess.

### 2.2. Experimental Evidences of Grafted Lignin

^1^H-NMR spectra (Figure S1) were recorded in order to elucidate the structural features of AL and AG materials (Table 1).

The ^1^H-NMR spectra evidenced three regions corresponding to hydrogen linked to: (i) aromatic carbons, (ii) side chain carbons and (iii) aliphatic carbons. The signals specific to carboxyl and aldehyde groups were also present at 10.86–9.63 ppm. The signal intensity of aromatic hydrogens increased in grafted lignin, and new aromatic signals were also observed in the region specific to aromatic protons (8.27–6.40 ppm). The signals at 6.3–4.00 ppm represented the H_α_ in β-*O*-4’ and β-1’ structures, H_α_ in β-5’ structures, H_α_ and H_β_ in β-*O*-4 structures and H_γ_ in β-β’ structures [37,39]. In addition, the spectra of the grafted lignin presented NH protons from polyaniline (5.01–4.99 ppm) [40]. The protons from methoxyl groups were observed in the region 3.90–3.20 ppm. DMSO-*d*_6_, used as a solvent, was determined at 2.5 ppm. The signals at 2.40–1.90 ppm corresponded to the aliphatic acetate protons, while the 1.60–0.60 ppm signals corresponded to aliphatic protons.

FTIR spectra were recorded for the identification of the structural changes in lignin before and after the grafting bioprocess (Figure S2). KL and KG samples were considered as representative examples. The identified bands are summarized in Table 2.

Grafting the lignin with aniline was also confirmed by FTIR spectra through the differences between the original (KL) and grated lignin (KG) (Figure S2). The intensity of the absorbance band at 3311 cm^−1^, specific for the O-H group, was weaker for KG compared with KL due to its involvement in the grafting process. The presence of aromatic rings in the range of 1600–1158 cm^−1^ was stronger for KG compared with KL, which was also an effect of grafting. New bands were observed at 1120 cm^−1^, 755 cm^−1^ and 698 cm^−1^. They were characteristic for –CO-NH–, –NH_2_ and –NH– groups, respectively. The new bands also confirmed the presence of aniline in the KG polymer.

### 2.3. Characterization of the Grafted Lignin

**Figure 5:**
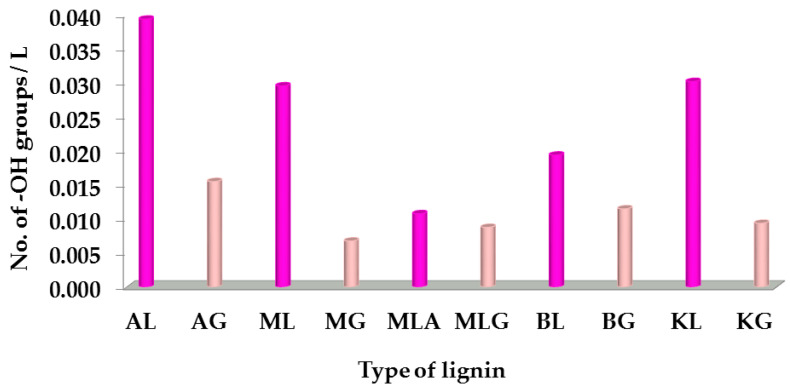
Differences between original and grafted lignins related to free –OH groups.

F-C analysis was performed for the determination of free –OH aromatic groups in the lignopolymeric structure (Figure 5). Grafted lignins were analyzed as well and the results were compared with those of original lignins.

As a general observation, all the lignins exhibited a decrease of free –OH groups after grafting, indicating the involvement of –OH in the grafting bioprocess. Large differences between original and grafted sample were observed for AL, ML and KL. AL and KL behavior was also confirmed based on UV-vis analysis (Figure 4). The large difference between ML and MG could be explained by intra oxi-polymerization of lignin instead of the amino-grafted bioprocess. Such a conclusion was also supported by GPC analysis (Table 3), which allowed to determine the molecular weight of the original and grafted polymers.

After grafting, the polymeric mass was enhanced (Table 3). The polydispersity index also increased, except for AL/AG. The higher molecular weight of the grafted polymer could be explained by the insertion of aniline as a monomer or an oligomer. The largest enhancement of the mass was registered for ML/MG as an effect of intra oxi-polymerization of lignin instead of lignin grafting. Increased values of PDI specific for all grafted lignins indicated a high heterogeneity of the grafted lignin population.

**Figure 6:**
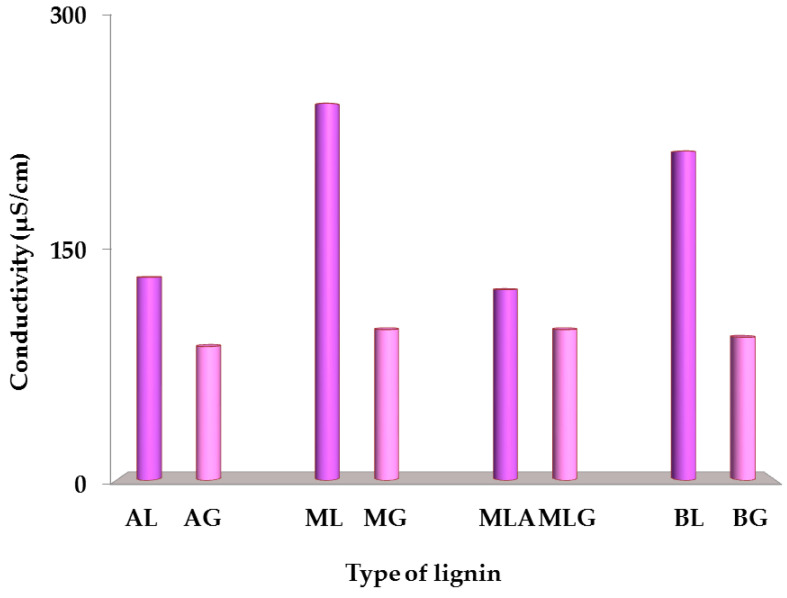
Conductivity measurements for original and grafted lignins.

The conductivity measurements were performed before and after the grafting bioprocess. (Figure 6). The initial lignins (AL, ML, MLA and BL) exhibited conductivities in the range of 124–245 µS/cm, while for the grafted lignins (AG, MG, MLG and BG), the conductivity suffered a strong decrease to 87–98 µS/cm. Such a modification was again a direct consequence of the variation of free –OH groups in the initial and grafted lignins. The grafted lignin exhibited a specific semiconductor property.

**Figure 7:**
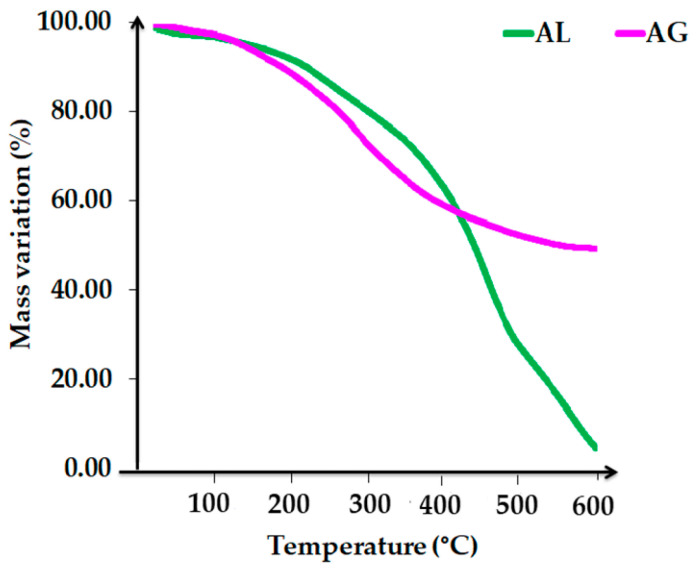
Thermogravimetric analysis (TGA) profiles of AL and AG.

The thermostability of the grafted and initial lignins was also investigated by TGA in the range of temperatures between 36 °C and 600 °C in nitrogen atmosphere (Figure 7).

The mass difference in the range of 36–100 °C indicated the loss of water, unspecifically entrapped in lignin structure (<8% for AL and 10–20% for AG). Further heating at 267–430 °C led to a larger mass loss, i.e., 40 wt% for the initial lignin and 46 wt% for the grafted polymer. The third range of temperature (430–600 °C) was associated with polymer degradation, accompanied by the elimination of CO_2_, NO_x_ and H_2_O. The grafted polymer was more stable than the original lignin. Thus, the grafting bioprocess induced an improvement in lignin thermostability.

The acidity/basicity of the lignin samples was evaluated using TPD-NH_3_/CO_2_ analysis (Table 4). The TPD-CO_2_ profile indicated a higher number of basic centers for AG compared with AL as a direct effect of aniline insertion. The TPD-NH_3_ measurements also confirmed the TPD-CO_2_ results (i.e., a decreased number of acidic centers attributed to the atoms with non-participating pair electrons playing the role of Lewis base sites, e.g., O and N).

**Figure 8:**
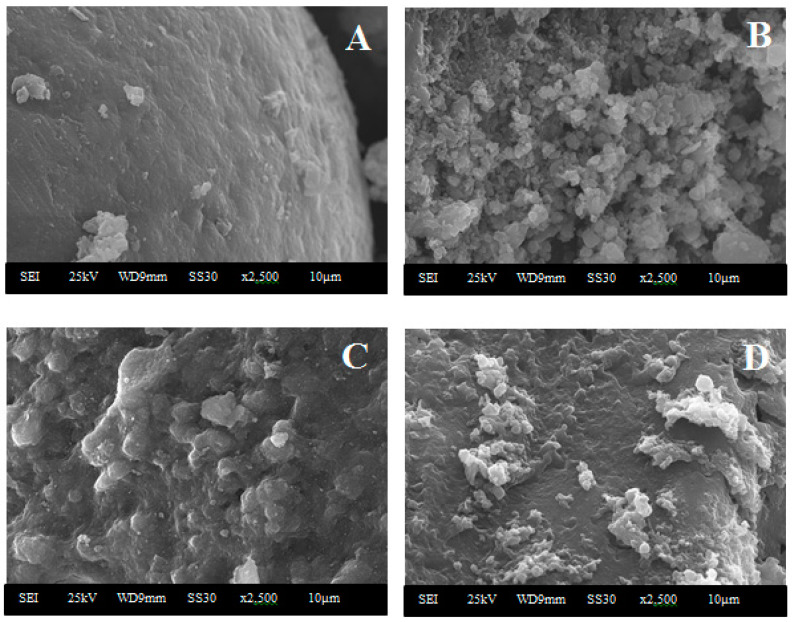
Scanning electron microscopy (SEM) images of (**A**) AL, (**B**) AG, (**C**) KL, (**D**) KG.

SEM images of AL-AG and KL-KG are presented in Figure 8. A smooth surface was evidenced for parent lignopolymers (AL and KL), while amino-grafted AG and KG lignopolymers showed a roughened morphology. The differences induced in the morphology of the grafted sample correlated to the type of lignin (AG polymeric layer looked more dense than KG polymeric layer).

## 3. Materials and Methods

### 3.1. Substances and Materials

Different types of lignin were tested in the grafting process (AL—alkali lignin provided by Sigma-Aldrich (St. Louis, MO, USA); ML—*Miscanthus* lignin extracted under acidic conditions; MLA—*Miscanthus* lignin extracted under alkali conditions; BL—beech lignin extracted by acid-H_2_O_2_ treatment; KL—lignin extracted using the Klason approach). Enzymatic screening for grafting lignin was achieved using four peroxidases from different sources: (i) horseradish peroxidase (HRP) type VI, essentially salt-free, lyophilized powder, ≥250 U mg^−1^ enzyme activity, solid purchased from Sigma-Aldrich (USA); (ii) 2-1B, versatile peroxidase originally from *Pleurotus eryngii*, expressed in *Saccharomyces cerevisiae*, 12.95 U mL^−1^ enzyme activity [41]; (iii) BB-8, mutant of versatile peroxidase from *Pleurotus eryngii*, expressed in *Saccharomyces cerevisiae*, 19.5 U mL^−1^ enzyme activity [42]; and (IV) PaDa, mutant of unspecific peroxidase from *Agrocybe aegerita*, expressed in *Saccharomyces cerevisiae*, 25.82 U mL^−1^ enzyme activity) [43]. All peroxidase mutants (2-1B, BB-8 and PaDa) were provided by Dr. Miguel Alcalde (Institute of Catalysis, CSIC, Madrid, Spain).

A phosphate buffer saline (PBS) was used as the buffer solution (10 mM, pH 7.4, consisting of 8 g NaCl, 0.2 g KCl, 1.43 g Na_2_HPO_4_ × 2H_2_O and 0.34 g KH_2_PO4 in 1 L distilled water. A 30 wt% solution of hydrogen peroxide (H_2_O_2_), methanol (MeOH), Na_2_CO_3_ and F-C reagent of analytical purity were purchased from Sigma-Aldrich.

### 3.2. Grafting Lignin with Aniline

**Scheme 2:**
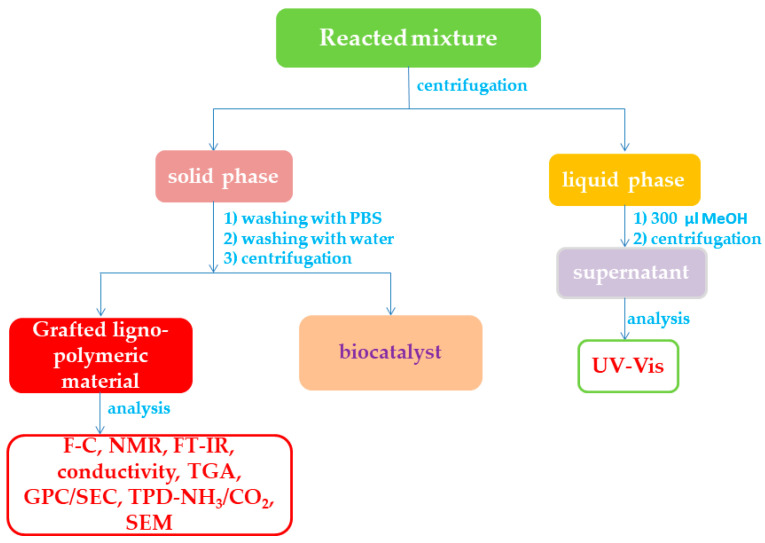
Pretreatment and analysis of the reacted mixture after the grafting bioprocess.

The grafting protocol of lignin is schematically presented in Scheme 2. The insertion of aniline in the lignin structure was performed using an enzymatic biocatalytic approach (Scheme 2). The reaction mixture contained 21.70 mg/mL lignin, 23.30 mM aniline, 192 mM H_2_O_2_ (30%, *w*/*w*), 11% (*v*/*v*) peroxidase enzyme corresponding to 2.5 U mg^−^^1^ enzyme activity and 22% MeOH in PBS (10 mM, pH 7.4). The reaction mixture was incubated for 24 h at 40 °C and 1000× *g* stirring ratio. After the reaction, the sample was centrifugated in order to separate the two phases, i.e., the grafted lignin as the solid phase and the reaction mixture left after the grafting process as the liquid phase. Lignin fraction was pretreated before characterization by washing two times with PBS and once with water. MeOH (300 μL) was added to the liquid phase in order to precipitate the enzyme. After centrifugation, the enzyme biocatalyst was recovered and stored in a PBS solution. Grafted lignopolymer was further characterized using F-C, NMR, FTIR, GPC, TPD-NH_3_/CO_2_ and SEM techniques. The supernatant was analyzed based on UV-vis analysis.

### 3.3. Spectrophotometric Analysis

The lignin derivatization with aniline was evaluated following the consumption of aniline from the reaction mixture. Thus, the UV-vis analysis of the liquid phase of the reaction mixture was performed before and after biocatalysis. The absorbance of the samples was read at 280 nm with a Specord 250 (Analytik Jena, Jena, Germany) instrument. Additionally, the free –OH groups on the aromatic ring were identified based on the F-C analysis [44]. The protocol was adapted for the investigation of the lignopolymer (Scheme 2). A 20 μL sample (10 mg/mL of lignopolymer in MeOH) was dispersed in 1.58 mL distilled water. The resulted suspension was mixed with 100 μL F-C reagent and 300 μL saturated solution of sodium carbonate as the supporting medium for adjusting the solution pH to a basic value. The mixture was incubated for 2 h in the dark and after that, the mixture absorbance was read at 765 nm. A calibration curve using the F-C reagent was determined for coniferyl alcohol in MeOH (0–0.01 M coniferyl alcohol) as a standard.

### 3.4. Characterization of Grafted Lignin with Aniline

FTIR spectra of original and grafted polymers were recorded using a Spectrum Two FTIR spectrometer (Perkin Elmer, Hamburg, Germany) equipped with a total attenuated reflectance cell in the range of 8300–350 cm^−1^. Fifty scans were collected with a resolution of 4 cm^−1^ in the range of 4000–400 cm^−1^.

Polymeric materials (original and grafted lignins) were evaluated based on size exclusion chromatography, using an Infinity (Model 1260, Agilent Technologies, Waldbronn, Germany) instrument with two columns (Zorbax PSM, 60-S, 6.5 mm × 250 mm, 5 mm and Polargel-M, 300 mm × 7.5 mm) and a multidetection system (260 GPC with RID (Refractive Index Detector), LSD (Light Scattering Detector) and VSD (Viscometer Detector)). The chromatographic separation was performed with 1 mL/min flow rate of tetrahydrofurane mobile phase, using 10 μL injection volume, at 35 °C and 40 °C of detector and column temperature, respectively. For SEC calibration curve, polystyrene standards with Mw in the range of 162–20,000 g/mol were used. Mw, Mn and PD of polymers were determined using an Agilent GPC (Version 1.1, Agilent Technologies) software. Taking into consideration that lignin can form aggregates (hydrogen bonding, van der Waals attraction of polymer chains [45]), the samples were acetylated before the analysis in order to avoid noncovalent interactions. The derivatization protocol comprised mixing 1.5 mL pyridine and 1.5 mL acetic anhydride with the sample and stirring the mixture for 24 h at room temperature. The resultant mixture (0.5 mL) was diluted with 1 mL ethanol, followed by evaporation at 70 °C. Diethyl ether (1 mL) and chloroform (1 mL) were added to the recovered solid for 1 h in a vortex at room temperature. After evaporation at 70 °C, the samples were dissolved in 1 mL THF. The resultant mixture was filtered and analyzed by GPC.

The NMR spectroscopy was used to investigate the lignin structure before and after the grafting process. ^1^H-NMR spectra were recorded on a Bruker Advance III Ultrashield Plus 500 MHz spectrometer (Leipzig, Germany), operating at 11.74 T, corresponding to the resonance frequency of 500.13 MHz for the ^1^H nucleus, equipped with a direct detection four-nuclei probe head and field gradients on the *z*-axis. The samples were dissolved in DMSO-*d*_6_ and recorded at 25 °C.

The thermogravimetric analysis (TGA) was performed with a Shimadzu instrument (Istanbul, Kurkey) (SDT Q600) in order to determinate the thermostability of the grafted lignin in relation to the original one. The analysis was carried out at increasing temperatures at a rate of 10 °C min^−1^ in the range of 36–600 °C under nitrogen atmosphere. A maximum of 12 mg of each sample was analyzed in aluminum capsules.

The electric conductivity of the original and grafted lignins was established using a WTW 340i conductivity meter (Vienna, Austria). The sample was dispersed in 1 mL MeOH. The conductivity values were expressed as µS/cm.

Temperature-programmed desorption of ammonia (TPD-NH_3_) and carbon dioxide (TPD-CO_2_) was performed using a Micromeritics Chemisorb 2750 instrument (Unterschleissheim, Germany) in order to evaluate the total acidity/basicity of the lignopolymeric material before and after the grafting bioprocess. First, the sample was heated to 120 °C with 5 °C min^−1^ for 30 min, in 30 mL highly pure helium flow in order to remove all the impurities. Then, it was cooled down to room temperature in helium flow. The adsorption of NH_3_/CO_2_ was carried out under ambient conditions for 1 h in a flow of 10% NH_3_/CO_2_ in helium (30 mL min^−1^). For NH_3_/CO_2_ desorption, the sample was heated up to 170 °C at a heating rate of 5 °C min^−1^.

Scanning electron microscopy (SEM) investigations were performed under high vacuum conditions. Freeze-dried particles were examined using a Jeol instrument (Peabody, MA, USA) (JSM-6610LV). The pretreatment of the samples followed the dispersion of the particles in EtOH solution (30%) and deposition of 10 µL suspension on the microscopic blade covered with a gold layer. After EtOH evaporation at room temperature, prepared blades were dried in vacuum followed by metal coating using a sputter coater (Jeol auto fine coater, JFC-1300, Peabody, MA, USA).

## 4. Conclusions

Grafting lignopolymers following an enzymatic approach was developed using HRP as the biocatalyst and H_2_O_2_ as the oxidation reagent. Aniline insertion was performed into the lignin polymeric structure as a grafted monomer. This corresponded to a decrease of the free –OH groups in the grafted lignin mainly as a direct consequence of aniline insertion (AL and KL). The grafting bioprocess was confirmed by several techniques such as ^1^H-NMR and FTIR, indicating the successful insertion of aniline into the lignin structure. Grafted lignin exhibited a larger polymeric mass, semiconductor property, higher thermostability and basicity compared to the parent lignin. Very importantly, the developed grafting bioprocess allowed to produce an amino-functionalized lignin with control of the properties.

As a general remark, the developed biografting process allowed to produce derivatized lignopolymers with grafted amino groups. In this way, the chemical reactivity of the lignin structure was enhanced, giving more alternatives for advanced lignin derivatization. The resultant grafted lignin exhibited suitable characteristics for industrial applications, such as ion-exchange resins, cationic surfactants, flocculants, coagulants and heavy metal adsorbents. Another future application that is being developed at the lab scale for the moment is the use of lignin as a support for protein immobilization.

Additionally, the biografting process offers a “green” alternative for lignin derivatization compared with the conventional chemical routes. The main advantages of the developed process are: (i) eco-friendly strategy, (ii) requires low biocatalyst concentration, (iii) avoids the use of conventional (metal) catalyst and (iv) limits the involvement of the organic solvent. The use of immobilized enzyme biocatalysts in the future will make them more cost-efficient and attractive candidates for industrial applications. The developed biografting process is also a promising alternative for improving lignin exploitation in the industrial area.

## Figures and Tables

**Table 1:** Chemical shifts assignments in ^1^H-NMR spectra of AL and AG.

Signal δ_H_/ppm	Assignments
10.86–9.63	Carboxyl and aldehyde groups
8.27–7.00	Aromatic protons in polyaniline/copolymers lignin-aniline (only in derivatized lignin)
7.60–7.20	Aromatic protons in hydroxyphenyl units (H)
7.20–6.80	Aromatic protons in guaiacyl units (G)
6.8–6.40	Aromatic protons in syringyl units (S)
6.3–5.7	H_α_ in β-O-4’ and β-1’ structures
5.70–5.10	H_α_ in β-5’ structures
5.01–4.99	NH protons from polyaniline (only in grafted lignin with aniline)
4.90–4.00	H_α_ and H_β_ in β-*O*-4 structures and H_γ_ in β-β’ structures
3.90–3.20	Methoxyl protons
2.40–1.90	Aliphatic acetate protons
1.60–0.60	Aliphatic protons

**Table 2:** Specific bands of original and grafted lignins.

Assignment	FTIR Band (cm^−1^)	
	KL	KG
O-H stretching	3429	3311
C-H stretching	2953	2946
C=O stretching	1730	1734
Aryl ring stretching, symmetric	1601	1599
Aryl ring stretching, asymmetric	1516	1505
C-H deformation, asymmetric	1464	1460
C-C of S-unit	1334	1330
C-O of S-unit	1275	1274
G-unit and C=O stretching vibration	1221	1223
C-H in plane deformation with G-unit	1163	1158
–CO-NH stretching vibration	-	1120
C-O stretching	1044	1035
C-H deformation of out-of-plane, aromatic ring	848	845
–NH_2_ deformation vibration	-	755
N-H wagging vibration	-	698

**Table 3:** Comparative gel permeation chromatography (GPC) data for initial and grafted lignins.

Sample	Mw	Mn	PDI
AL	14,054	6319	2.224
AG	22,374	13,229	1.691
ML	6890	5880	1.172
MG	22,253	5814	3.828
KL	85,435	84,777	1.008
KG	106,026	91,987	1.153

**Table 4:** Acidity/basicity of lignin before and after the grafting bioprocess.

Sample	Basicity (µmol/g)	Acidity (µmol/g)
AL	5.89	10.88
AG	16.98	4.49

## Supplementary Materials

The following are available online, Figure S1: ^1^H-NMR spectra of (A) AL (unmodified lignin) and (B) AG (grafted lignin), Figure S2: FTIR spectra of KL and corresponding grafted polymer (KG).
