# Supplementary material for: Biocatalytic Strategy for Grafting Natural Lignin with Aniline
Source: Molecules. 2020 Oct 24;25(21):4921. doi: 10.3390/molecules25214921 (PMC7662662; doi:10.3390/molecules25214921)
Supplement: Supplementary file 1 [file molecules-25-04921-s001.pdf]

molecules

Supplementary Materials

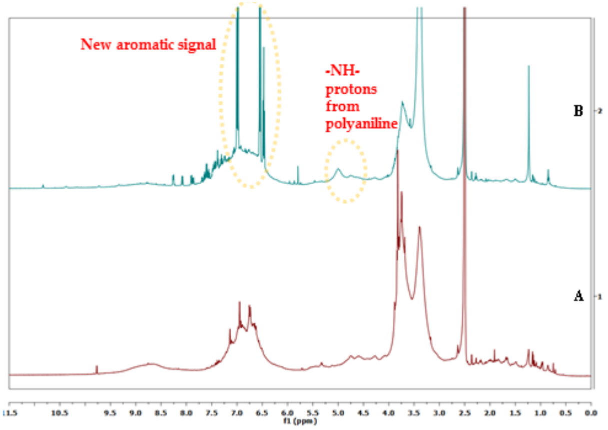

Figure S1: ^{1}H-NMR spectra of: A – AL and B – AG.

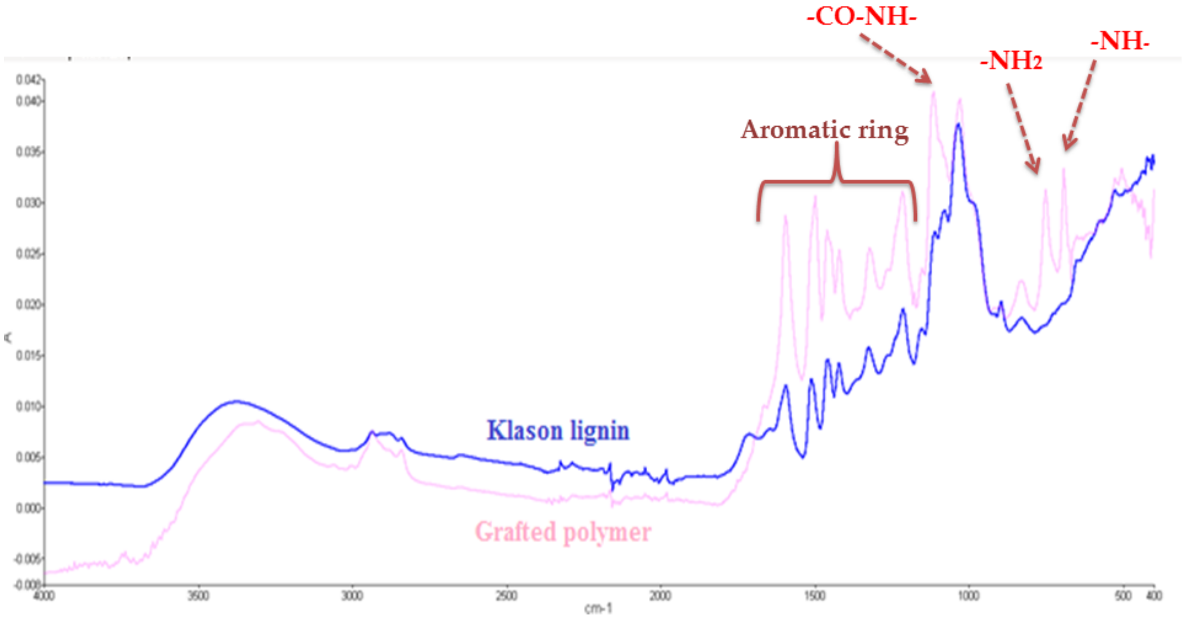

Figure S2: FTIR spectra of KL and corresponding grafted polymer (KG).
